# Tris(nitrato-κ^2^ *O*,*O*′)bis­(1,10-phenanthroline-κ^2^ *N*,*N*′)holmium(III)

**DOI:** 10.1107/S1600536808020655

**Published:** 2008-07-09

**Authors:** Eny Kusrini, Muhammad Idiris Saleh, Reza Kia, Hoong-Kun Fun

**Affiliations:** a School of Chemical Sciences, Universiti Sains Malaysia, 11800 USM, Penang, Malaysia; b X-ray Crystallography Unit, School of Physics, Universiti Sains Malaysia, 11800 USM, Penang, Malaysia

## Abstract

In the title compound, [Ho(NO_3_)_3_(C_12_H_8_N_2_)_2_], the ten-coordinate Ho^III^ ion is chelated by four N atoms from two phenanthroline (phen) ligands and six O atoms from three bidentate nitrate groups. The environment around the Ho atom can be described as a distorted bicapped square anti­prism. Two phenanthroline ligands form a dihedral angle of 43.72 (13)°. Short inter­molecular distances between the centroids of the six-membered rings [3.6887 (14)–3.8374 (16) Å] indicate the existence of π–π inter­actions, which link the mol­ecules into stacks extended in the [10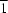] direction. The crystal packing is further stabilized by weak inter­molecular C—H⋯O hydrogen bonds.

## Related literature

For related literature on hydrogen bond motifs, see: Bernstein *et al.* (1995 ▶). For bond-length data, see: Allen *et al.* (1987 ▶). For related literature, see, for example: Frechette *et al.* (1992 ▶); Lin & Feng (2003 ▶); Zheng *et al.* (2001 ▶); Antsyshkina *et al.* (2002 ▶); Sadikov *et al.* (2006*a* ▶,*b* ▶); Rybakov *et al.* (1991 ▶); Wei *et al.* (2002 ▶); Kepert *et al.* (1996 ▶); Liu *et al.* (2007 ▶); Xu *et al.*, (2005 ▶).
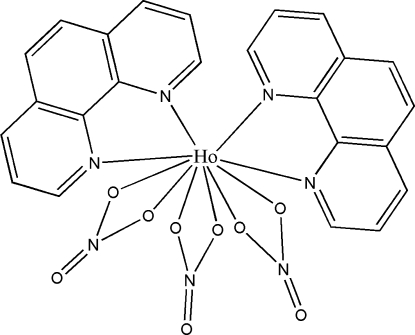


## Experimental

### 

#### Crystal data

[Ho(NO_3_)_3_(C_12_H_8_N_2_)_2_]
*M* *_r_* = 711.37
Monoclinic, 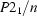

*a* = 11.0049 (2) Å
*b* = 17.7710 (3) Å
*c* = 12.9332 (2) Å
β = 100.483 (1)°
*V* = 2487.10 (7) Å^3^
*Z* = 4
Mo *K*α radiation
μ = 3.25 mm^−1^
*T* = 100.0 (1) K
0.34 × 0.19 × 0.11 mm

#### Data collection

Bruker SMART APEXII CCD area-detector diffractometer
Absorption correction: multi-scan (*SADABS*; Bruker, 2005 ▶) *T*_min_ = 0.548, *T*_max_ = 0.727
39049 measured reflections
7244 independent reflections
6059 reflections with *I* > 2σ(*I*)
*R*_int_ = 0.049

#### Refinement

*R*[*F* ^2^ > 2σ(*F* ^2^)] = 0.031
*wR*(*F* ^2^) = 0.070
*S* = 1.05
7244 reflections
371 parameters
H-atom parameters constrained
Δρ_max_ = 3.31 e Å^−3^
Δρ_min_ = −1.08 e Å^−3^

### 

Data collection: *APEX2* (Bruker, 2005 ▶); cell refinement: *APEX2*; data reduction: *SAINT* (Bruker, 2005 ▶); program(s) used to solve structure: *SHELXTL* (Sheldrick, 2008 ▶); program(s) used to refine structure: *SHELXTL*; molecular graphics: *SHELXTL*; software used to prepare material for publication: *SHELXTL* and *PLATON* (Spek, 2003 ▶).

## Supplementary Material

Crystal structure: contains datablocks global, I. DOI: 10.1107/S1600536808020655/cv2423sup1.cif

Structure factors: contains datablocks I. DOI: 10.1107/S1600536808020655/cv2423Isup2.hkl

Additional supplementary materials:  crystallographic information; 3D view; checkCIF report

## Figures and Tables

**Table 1:** Selected interatomic distances (Å)

*Cg*1⋯*Cg*3	3.8375 (16)
*Cg*2⋯*Cg*4^i^	3.7202 (14)
*Cg*2⋯*Cg*6^i^	3.7641 (15)
*Cg*4⋯*Cg*5^ii^	3.6887 (14)

Symmetry codes: (i) 
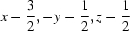
; (ii) 
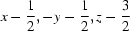
. *Cg*1, *Cg*2, *Cg*3, *Cg*4, *Cg*5, and *Cg*6 are the centroids of atoms N1/C1–C4/C12,  N2/C7–C11, N3/C13–C16/C24, N4/C19–C23, C4–C7/C11–C12, and  C16–C19/C23–C24, respectively.

**Table 2:** Hydrogen-bond geometry (Å, °)

*D*—H⋯*A*	*D*—H	H⋯*A*	*D*⋯*A*	*D*—H⋯*A*
C3—H3*A*⋯O8^iii^	0.93	2.53	3.422 (3)	161
C9—H9*A*⋯O3^iv^	0.93	2.55	3.453 (3)	164
C21—H21*A*⋯O3^v^	0.93	2.43	3.201 (3)	140

Symmetry codes: (iii) 
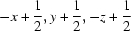
; (iv) 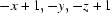; (v) 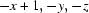.

**Fig. 1.**
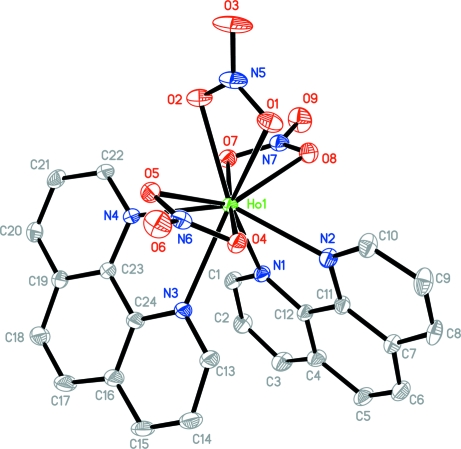
The molecular structure of (I), showing 50% probability displacement ellipsoids and the atomic numbering. The H atoms have been omitted for clarity.

## supplementary crystallographic information

### Comment

1,10-Phenanthroline (Phen) has experienced an increasingly important role in the field of supramolecular chemistry as a ligand and sensitizer in different lanthanide complexes. The series of lanthanide nitrate complexes (La, Ce, Pr, Nd, Sm, Eu, Gd, Tb, Dy, Yb and Lu) with the Phen ligand have previously been reported (Frechette *et al.*, 1992; Lin & Feng, 2003; Zheng *et al.*, 2001; Antsyshkina *et al.*, 2002; Sadikov *et al.*, 2006*a*,b; Rybakov *et al.*, 1991; Wei *et al.*, 2002; Kepert *et al.*, 1996; Liu *et al.*, 2007], while crystal structure  of [Ho(Phen)_2_(NO_3_)_3_] complex has not been  reported in the literature. In these complexes, it is believed that the Phen ligand may displace the coordinated water molecules and increase the molecular rigidity to attain the high luminescence efficiency (Xu *et al.*, 2005). Almost all of the [Ln(Phen)_2_(NO_3_)_3_] {Ln = La, Ce, Pr, Nd, Sm, Eu, Tb, Dy, Yb, Lu} complexes were crystallized in the monoclinic space group *C*2/*c*. In our study, the [Ho(Phen)_2_(NO_3_)_3_] complex was obtained from a solution mixture containing 1,10-phenanthroline, tetraethylene glycol and holmium nitrate.

In the title compound (Fig. 1), four nitrogen atoms (from two phen ligands; N1, N2, N3, and N4) and six oxygen atoms from three bidentate nitrate groups (O1, O2; O4, O5; O7, O8) are coordinated to the central Ho^III^ ion. Bond lengths and angles have normal values (Allen *et al.*, 1987). The dihedral angle between the two phenanthroline ligands is 43.72 (13) °.

In the crystal structure, short intermolecular distances between the centroids of six-membered rings (Table 1) prove an existence of π-π interactions, which link the molecules into stacks extended in direction [10-1]. The crystal packing is further stabilized by the weak intermolecular C—H···O hydrogen bonds (Table 2).

### Experimental

The title compound was prepared by the reaction of tetraethylene glycol (0.207 g, 1.07 mmol), 1,10-phenanthroline (0.179, 1 mmol) and holmium nitrate (0.442 g, 1 mmol) in 20 ml H_2_O. The solution was heated for 1 h at 60°C. The solution was filtrated and was covered with an aluminium foil to enable slow evaporation at room temperature. Brown crystals were obtained after 5 months with a yield of 80%. Anal. Calc. for. [Ho(Phen)_2_(NO_3_)_3_]: C, 40.49; H, 2.25; N, 13.78. Found: C, 37.93; H, 2.25; N, 12.35%.

### Refinement

The geometrically constrained hydrogen atoms were placed in calculated positions (C-H 0.93 Å) and refined as riding with *U*_iso_(H) = 1.2 *U*_eq_(C). The highest residual peak [3.31 eÅ^-3^] is located 0.83 Å from Ho1 and the deepest hole [-1.08 eÅ^-3^] is located 2.04 Å from C15.

### Crystal data

**Table:**

[Ho(NO_3_)_3_(C_12_H_8_N_2_)_2_]	*F*_000_ = 1392
*M**_r_* = 711.37	*D*_x_ = 1.900 Mg m^−^^3^
Monoclinic, *P*2_1_/*n*	Mo *K*α radiation λ = 0.71073 Å
Hall symbol: -P 2yn	Cell parameters from 9346 reflections
*a* = 11.0049 (2) Å	θ = 2.2–32.1º
*b* = 17.7710 (3) Å	µ = 3.25 mm^−^^1^
*c* = 12.9332 (2) Å	*T* = 100.0 (1) K
β = 100.4830 (10)º	Block, brown
*V* = 2487.10 (7) Å^3^	0.34 × 0.19 × 0.11 mm
*Z* = 4	

### Data collection

**Table:**

Bruker SMART APEXII CCD area-detector diffractometer	7244 independent reflections
Radiation source: fine-focus sealed tube	6059 reflections with *I* > 2σ(*I*)
Monochromator: graphite	*R*_int_ = 0.049
*T* = 100.0(1) K	θ_max_ = 30.0º
φ and ω scans	θ_min_ = 2.0º
Absorption correction: multi-scan(SADABS; Bruker, 2005)	*h* = −15→15
*T*_min_ = 0.548, *T*_max_ = 0.727	*k* = −25→25
39049 measured reflections	*l* = −18→18

### Refinement

**Table:**

Refinement on *F*^2^	Secondary atom site location: difference Fourier map
Least-squares matrix: full	Hydrogen site location: inferred from neighbouring sites
*R*[*F*^2^ > 2σ(*F*^2^)] = 0.031	H-atom parameters constrained
*wR*(*F*^2^) = 0.070	*w* = 1/[σ^2^(*F*_o_^2^) + (0.0236*P*)^2^ + 4.9031*P*] where *P* = (*F*_o_^2^ + 2*F*_c_^2^)/3
*S* = 1.05	(Δ/σ)_max_ = 0.004
7244 reflections	Δρ_max_ = 3.31 e Å^−^^3^
371 parameters	Δρ_min_ = −1.08 e Å^−^^3^
Primary atom site location: structure-invariant direct methods	Extinction correction: none

### Special details

**Table:**

Experimental. The low-temperature data was collected with the Oxford Cyrosystem Cobra low-temperature attachment.
Geometry. All e.s.d.'s (except the e.s.d. in the dihedral angle between two l.s. planes) are estimated using the full covariance matrix. The cell e.s.d.'s are taken into account individually in the estimation of e.s.d.'s in distances, angles and torsion angles; correlations between e.s.d.'s in cell parameters are only used when they are defined by crystal symmetry. An approximate (isotropic) treatment of cell e.s.d.'s is used for estimating e.s.d.'s involving l.s. planes.
Refinement. Refinement of *F*^2^ against ALL reflections. The weighted *R*-factor *wR* and goodness of fit *S* are based on *F*^2^, conventional *R*-factors *R* are based on *F*, with *F* set to zero for negative *F*^2^. The threshold expression of *F*^2^ > σ(*F*^2^) is used only for calculating *R*-factors(gt) *etc*. and is not relevant to the choice of reflections for refinement. *R*-factors based on *F*^2^ are statistically about twice as large as those based on *F*, and *R*- factors based on ALL data will be even larger.

### Fractional atomic coordinates and isotropic or equivalent isotropic displacement parameters (Å^2^)

**Table:**

	*x*	*y*	*z*	*U*_iso_*/*U*_eq_	
Ho1	0.488774 (10)	0.175637 (6)	0.238530 (8)	0.01342 (3)	
O1	0.51495 (18)	0.04947 (10)	0.31894 (15)	0.0232 (4)	
O2	0.48440 (18)	0.05435 (10)	0.14900 (15)	0.0233 (4)	
O3	0.5190 (2)	−0.05374 (11)	0.22803 (19)	0.0394 (6)	
O4	0.66600 (16)	0.16813 (10)	0.39018 (13)	0.0183 (4)	
O5	0.70134 (16)	0.13574 (10)	0.23748 (13)	0.0188 (4)	
O6	0.84805 (18)	0.12616 (12)	0.37481 (15)	0.0276 (5)	
O7	0.30817 (17)	0.16390 (10)	0.08994 (13)	0.0195 (4)	
O8	0.28298 (16)	0.12639 (10)	0.24356 (13)	0.0200 (4)	
O9	0.12801 (18)	0.12176 (13)	0.11134 (16)	0.0312 (5)	
N1	0.35309 (19)	0.29166 (11)	0.22497 (15)	0.0160 (4)	
N2	0.4282 (2)	0.21458 (12)	0.40627 (16)	0.0173 (4)	
N3	0.6131 (2)	0.29709 (11)	0.25716 (15)	0.0171 (4)	
N4	0.54335 (19)	0.22274 (11)	0.07176 (15)	0.0156 (4)	
N5	0.5064 (2)	0.01456 (12)	0.23142 (19)	0.0238 (5)	
N6	0.74234 (19)	0.14297 (12)	0.33588 (16)	0.0177 (4)	
N7	0.2359 (2)	0.13717 (12)	0.14692 (17)	0.0189 (5)	
C1	0.3141 (2)	0.32922 (14)	0.13647 (18)	0.0186 (5)	
H1A	0.3194	0.3060	0.0730	0.022*	
C2	0.2651 (3)	0.40222 (14)	0.1339 (2)	0.0221 (6)	
H2A	0.2393	0.4265	0.0700	0.027*	
C3	0.2558 (3)	0.43717 (15)	0.2259 (2)	0.0232 (6)	
H3A	0.2261	0.4862	0.2254	0.028*	
C4	0.2916 (2)	0.39856 (14)	0.3218 (2)	0.0200 (5)	
C5	0.2779 (3)	0.42963 (15)	0.4216 (2)	0.0236 (6)	
H5A	0.2468	0.4781	0.4248	0.028*	
C6	0.3098 (3)	0.38902 (15)	0.5111 (2)	0.0229 (6)	
H6A	0.2995	0.4100	0.5749	0.027*	
C7	0.3588 (2)	0.31498 (14)	0.50970 (18)	0.0181 (5)	
C8	0.3894 (2)	0.26962 (16)	0.60012 (19)	0.0221 (6)	
H8A	0.3786	0.2878	0.6653	0.027*	
C9	0.4350 (3)	0.19881 (16)	0.5915 (2)	0.0240 (6)	
H9A	0.4528	0.1677	0.6502	0.029*	
C10	0.4544 (2)	0.17361 (15)	0.49320 (19)	0.0210 (5)	
H10A	0.4874	0.1258	0.4886	0.025*	
C11	0.3779 (2)	0.28392 (14)	0.41332 (18)	0.0159 (5)	
C12	0.3402 (2)	0.32587 (14)	0.31716 (18)	0.0161 (5)	
C13	0.6460 (3)	0.33423 (14)	0.34675 (19)	0.0208 (5)	
H13A	0.6407	0.3094	0.4091	0.025*	
C14	0.6882 (3)	0.40881 (15)	0.3528 (2)	0.0267 (6)	
H14A	0.7088	0.4327	0.4176	0.032*	
C15	0.6986 (3)	0.44575 (15)	0.2625 (2)	0.0282 (6)	
H15A	0.7247	0.4956	0.2648	0.034*	
C16	0.6691 (3)	0.40757 (15)	0.1656 (2)	0.0234 (6)	
C17	0.6835 (3)	0.44123 (16)	0.0676 (2)	0.0300 (7)	
H17A	0.7114	0.4906	0.0669	0.036*	
C18	0.6571 (3)	0.40208 (15)	−0.0241 (2)	0.0267 (6)	
H18A	0.6685	0.4245	−0.0866	0.032*	
C19	0.6119 (2)	0.32673 (14)	−0.02508 (18)	0.0185 (5)	
C20	0.5850 (2)	0.28446 (15)	−0.11786 (19)	0.0206 (5)	
H20A	0.5976	0.3047	−0.1814	0.025*	
C21	0.5399 (2)	0.21275 (15)	−0.11353 (19)	0.0212 (6)	
H21A	0.5237	0.1834	−0.1740	0.025*	
C22	0.5182 (2)	0.18402 (14)	−0.01784 (19)	0.0185 (5)	
H22A	0.4848	0.1360	−0.0167	0.022*	
C23	0.5929 (2)	0.29317 (14)	0.06874 (18)	0.0163 (5)	
C24	0.6252 (2)	0.33318 (14)	0.16655 (18)	0.0173 (5)	

### Atomic displacement parameters (Å^2^)

**Table:**

	*U*^11^	*U*^22^	*U*^33^	*U*^12^	*U*^13^	*U*^23^
Ho1	0.01761 (5)	0.00975 (5)	0.01367 (5)	0.00001 (4)	0.00491 (4)	−0.00004 (4)
O1	0.0251 (9)	0.0166 (9)	0.0260 (9)	−0.0010 (8)	−0.0006 (8)	0.0047 (7)
O2	0.0262 (9)	0.0146 (8)	0.0272 (9)	0.0017 (8)	−0.0001 (8)	−0.0017 (7)
O3	0.0416 (13)	0.0101 (9)	0.0596 (14)	0.0017 (9)	−0.0090 (11)	−0.0001 (9)
O4	0.0231 (8)	0.0178 (8)	0.0151 (7)	−0.0001 (7)	0.0063 (7)	−0.0008 (6)
O5	0.0203 (8)	0.0203 (9)	0.0159 (8)	−0.0017 (7)	0.0034 (7)	−0.0014 (7)
O6	0.0204 (9)	0.0305 (10)	0.0299 (10)	0.0005 (8)	−0.0006 (8)	0.0013 (8)
O7	0.0238 (9)	0.0178 (9)	0.0173 (8)	0.0003 (7)	0.0047 (7)	−0.0004 (6)
O8	0.0219 (9)	0.0208 (9)	0.0169 (8)	0.0005 (7)	0.0029 (7)	0.0022 (7)
O9	0.0176 (9)	0.0412 (12)	0.0325 (10)	−0.0039 (9)	−0.0015 (8)	−0.0013 (9)
N1	0.0212 (10)	0.0132 (9)	0.0143 (9)	0.0015 (8)	0.0050 (8)	0.0002 (7)
N2	0.0213 (10)	0.0147 (9)	0.0163 (9)	0.0045 (8)	0.0051 (8)	0.0024 (7)
N3	0.0238 (10)	0.0140 (9)	0.0143 (9)	−0.0035 (8)	0.0058 (8)	−0.0007 (7)
N4	0.0181 (9)	0.0133 (9)	0.0160 (9)	−0.0008 (8)	0.0047 (8)	−0.0026 (7)
N5	0.0199 (11)	0.0126 (10)	0.0362 (12)	0.0009 (8)	−0.0025 (10)	−0.0012 (9)
N6	0.0181 (10)	0.0150 (10)	0.0196 (9)	−0.0033 (8)	0.0024 (8)	−0.0004 (8)
N7	0.0206 (10)	0.0146 (10)	0.0215 (10)	0.0014 (8)	0.0037 (8)	−0.0003 (8)
C1	0.0277 (12)	0.0157 (11)	0.0129 (10)	0.0032 (10)	0.0046 (9)	0.0027 (9)
C2	0.0313 (14)	0.0172 (12)	0.0186 (11)	0.0058 (11)	0.0061 (10)	0.0057 (9)
C3	0.0304 (14)	0.0140 (11)	0.0254 (12)	0.0085 (11)	0.0059 (11)	0.0026 (9)
C4	0.0258 (13)	0.0128 (11)	0.0217 (11)	0.0033 (10)	0.0054 (10)	−0.0001 (9)
C5	0.0351 (14)	0.0155 (12)	0.0222 (12)	0.0037 (11)	0.0104 (11)	−0.0034 (9)
C6	0.0313 (14)	0.0216 (13)	0.0167 (11)	0.0016 (11)	0.0071 (10)	−0.0029 (9)
C7	0.0203 (11)	0.0200 (12)	0.0146 (10)	0.0000 (10)	0.0046 (9)	−0.0013 (9)
C8	0.0257 (13)	0.0308 (14)	0.0111 (10)	0.0019 (11)	0.0065 (9)	0.0003 (9)
C9	0.0263 (13)	0.0305 (14)	0.0162 (11)	0.0079 (11)	0.0062 (10)	0.0071 (10)
C10	0.0253 (12)	0.0213 (12)	0.0175 (10)	0.0076 (11)	0.0067 (9)	0.0059 (10)
C11	0.0166 (11)	0.0164 (11)	0.0156 (10)	0.0014 (9)	0.0054 (9)	−0.0001 (8)
C12	0.0212 (11)	0.0142 (10)	0.0136 (9)	0.0005 (10)	0.0051 (8)	0.0002 (8)
C13	0.0299 (13)	0.0179 (12)	0.0154 (10)	−0.0038 (10)	0.0066 (10)	−0.0028 (9)
C14	0.0443 (16)	0.0189 (12)	0.0177 (11)	−0.0097 (12)	0.0076 (11)	−0.0057 (10)
C15	0.0467 (17)	0.0165 (12)	0.0228 (12)	−0.0122 (12)	0.0105 (12)	−0.0060 (10)
C16	0.0356 (14)	0.0149 (12)	0.0216 (11)	−0.0057 (11)	0.0107 (11)	−0.0016 (9)
C17	0.0513 (18)	0.0183 (12)	0.0232 (12)	−0.0137 (13)	0.0138 (12)	−0.0031 (10)
C18	0.0425 (16)	0.0207 (13)	0.0195 (11)	−0.0093 (12)	0.0126 (11)	0.0004 (10)
C19	0.0228 (12)	0.0188 (11)	0.0149 (10)	−0.0023 (10)	0.0063 (9)	−0.0008 (9)
C20	0.0231 (12)	0.0236 (13)	0.0163 (10)	−0.0006 (11)	0.0070 (9)	0.0007 (9)
C21	0.0259 (13)	0.0252 (13)	0.0127 (10)	−0.0008 (11)	0.0040 (9)	−0.0054 (9)
C22	0.0236 (12)	0.0152 (11)	0.0176 (10)	−0.0030 (10)	0.0058 (9)	−0.0043 (9)
C23	0.0189 (11)	0.0149 (11)	0.0159 (10)	−0.0012 (9)	0.0055 (9)	−0.0015 (8)
C24	0.0217 (11)	0.0156 (11)	0.0148 (10)	−0.0028 (10)	0.0039 (9)	−0.0005 (8)

### Geometric parameters (Å, °)

**Table:**

Ho1—O8	2.4398 (18)	C4—C12	1.404 (3)
Ho1—O2	2.4431 (18)	C4—C5	1.436 (4)
Ho1—O5	2.4468 (18)	C5—C6	1.356 (4)
Ho1—O1	2.4662 (18)	C5—H5A	0.9300
Ho1—N2	2.481 (2)	C6—C7	1.423 (4)
Ho1—N4	2.487 (2)	C6—H6A	0.9300
Ho1—O4	2.5053 (17)	C7—C8	1.410 (3)
Ho1—O7	2.5094 (17)	C7—C11	1.413 (3)
Ho1—N1	2.533 (2)	C8—C9	1.367 (4)
Ho1—N3	2.544 (2)	C8—H8A	0.9300
Ho1—N5	2.872 (2)	C9—C10	1.401 (4)
Ho1—N7	2.901 (2)	C9—H9A	0.9300
O1—N5	1.279 (3)	C10—H10A	0.9300
O2—N5	1.265 (3)	C11—C12	1.445 (3)
O3—N5	1.223 (3)	C13—C14	1.402 (4)
O4—N6	1.270 (3)	C13—H13A	0.9300
O5—N6	1.277 (3)	C14—C15	1.362 (4)
O6—N6	1.218 (3)	C14—H14A	0.9300
O7—N7	1.271 (3)	C15—C16	1.411 (4)
O8—N7	1.277 (3)	C15—H15A	0.9300
O9—N7	1.224 (3)	C16—C24	1.409 (3)
N1—C1	1.327 (3)	C16—C17	1.435 (4)
N1—C12	1.369 (3)	C17—C18	1.361 (4)
N2—C10	1.326 (3)	C17—H17A	0.9300
N2—C11	1.361 (3)	C18—C19	1.428 (4)
N3—C13	1.325 (3)	C18—H18A	0.9300
N3—C24	1.363 (3)	C19—C20	1.401 (3)
N4—C22	1.333 (3)	C19—C23	1.401 (3)
N4—C23	1.369 (3)	C20—C21	1.373 (4)
C1—C2	1.403 (3)	C20—H20A	0.9300
C1—H1A	0.9300	C21—C22	1.399 (3)
C2—C3	1.362 (4)	C21—H21A	0.9300
C2—H2A	0.9300	C22—H22A	0.9300
C3—C4	1.410 (3)	C23—C24	1.438 (3)
C3—H3A	0.9300		
			
Cg1···Cg3	3.8375 (16)	Cg2···Cg6^i^	3.7641 (15)
Cg2···Cg4^i^	3.7202 (14)	Cg4···Cg5^ii^	3.6887 (14)
			
O8—Ho1—O2	75.99 (6)	O3—N5—Ho1	177.36 (19)
O8—Ho1—O5	142.12 (6)	O2—N5—Ho1	57.74 (12)
O2—Ho1—O5	71.22 (6)	O1—N5—Ho1	58.85 (12)
O8—Ho1—O1	72.57 (6)	O6—N6—O4	122.4 (2)
O2—Ho1—O1	52.30 (6)	O6—N6—O5	121.7 (2)
O5—Ho1—O1	72.57 (6)	O4—N6—O5	115.92 (19)
O8—Ho1—N2	71.23 (6)	O6—N6—Ho1	177.16 (17)
O2—Ho1—N2	132.47 (7)	O4—N6—Ho1	59.28 (11)
O5—Ho1—N2	119.67 (6)	O5—N6—Ho1	56.67 (11)
O1—Ho1—N2	85.08 (7)	O9—N7—O7	122.2 (2)
O8—Ho1—N4	121.04 (6)	O9—N7—O8	121.8 (2)
O2—Ho1—N4	82.70 (6)	O7—N7—O8	116.0 (2)
O5—Ho1—N4	73.19 (6)	O9—N7—Ho1	177.83 (18)
O1—Ho1—N4	130.06 (7)	O7—N7—Ho1	59.57 (11)
N2—Ho1—N4	144.11 (7)	O8—N7—Ho1	56.46 (12)
O8—Ho1—O4	123.66 (6)	N1—C1—C2	123.2 (2)
O2—Ho1—O4	105.63 (6)	N1—C1—H1A	118.4
O5—Ho1—O4	51.68 (6)	C2—C1—H1A	118.4
O1—Ho1—O4	66.85 (6)	C3—C2—C1	119.4 (2)
N2—Ho1—O4	67.99 (6)	C3—C2—H2A	120.3
N4—Ho1—O4	114.86 (6)	C1—C2—H2A	120.3
O8—Ho1—O7	51.75 (6)	C2—C3—C4	119.6 (2)
O2—Ho1—O7	67.62 (6)	C2—C3—H3A	120.2
O5—Ho1—O7	126.62 (6)	C4—C3—H3A	120.2
O1—Ho1—O7	105.24 (6)	C12—C4—C3	117.2 (2)
N2—Ho1—O7	113.04 (6)	C12—C4—C5	119.7 (2)
N4—Ho1—O7	69.29 (6)	C3—C4—C5	123.1 (2)
O4—Ho1—O7	172.06 (6)	C6—C5—C4	120.6 (2)
O8—Ho1—N1	75.84 (6)	C6—C5—H5A	119.7
O2—Ho1—N1	136.45 (6)	C4—C5—H5A	119.7
O5—Ho1—N1	141.96 (6)	C5—C6—C7	121.4 (2)
O1—Ho1—N1	142.31 (7)	C5—C6—H6A	119.3
N2—Ho1—N1	65.46 (6)	C7—C6—H6A	119.3
N4—Ho1—N1	84.16 (7)	C8—C7—C11	117.2 (2)
O4—Ho1—N1	117.56 (6)	C8—C7—C6	123.5 (2)
O7—Ho1—N1	68.89 (6)	C11—C7—C6	119.3 (2)
O8—Ho1—N3	141.99 (7)	C9—C8—C7	119.7 (2)
O2—Ho1—N3	139.24 (7)	C9—C8—H8A	120.2
O5—Ho1—N3	75.40 (6)	C7—C8—H8A	120.2
O1—Ho1—N3	135.39 (6)	C8—C9—C10	119.2 (2)
N2—Ho1—N3	84.61 (7)	C8—C9—H9A	120.4
N4—Ho1—N3	65.53 (6)	C10—C9—H9A	120.4
O4—Ho1—N3	69.04 (6)	N2—C10—C9	123.2 (2)
O7—Ho1—N3	118.72 (6)	N2—C10—H10A	118.4
N1—Ho1—N3	67.42 (7)	C9—C10—H10A	118.4
O8—Ho1—N5	73.26 (6)	N2—C11—C7	122.7 (2)
O2—Ho1—N5	25.97 (6)	N2—C11—C12	117.8 (2)
O5—Ho1—N5	68.94 (6)	C7—C11—C12	119.5 (2)
O1—Ho1—N5	26.35 (6)	N1—C12—C4	123.2 (2)
N2—Ho1—N5	109.67 (7)	N1—C12—C11	117.5 (2)
N4—Ho1—N5	106.22 (7)	C4—C12—C11	119.3 (2)
O4—Ho1—N5	85.66 (6)	N3—C13—C14	123.7 (2)
O7—Ho1—N5	86.64 (6)	N3—C13—H13A	118.2
N1—Ho1—N5	148.39 (6)	C14—C13—H13A	118.2
N3—Ho1—N5	144.14 (7)	C15—C14—C13	119.1 (2)
O8—Ho1—N7	25.87 (6)	C15—C14—H14A	120.4
O2—Ho1—N7	70.36 (6)	C13—C14—H14A	120.4
O5—Ho1—N7	140.94 (6)	C14—C15—C16	119.3 (2)
O1—Ho1—N7	89.30 (6)	C14—C15—H15A	120.4
N2—Ho1—N7	91.85 (7)	C16—C15—H15A	120.4
N4—Ho1—N7	95.18 (6)	C24—C16—C15	117.8 (2)
O4—Ho1—N7	149.26 (6)	C24—C16—C17	119.5 (2)
O7—Ho1—N7	25.89 (6)	C15—C16—C17	122.7 (2)
N1—Ho1—N7	69.75 (6)	C18—C17—C16	121.1 (3)
N3—Ho1—N7	134.29 (6)	C18—C17—H17A	119.5
N5—Ho1—N7	79.49 (6)	C16—C17—H17A	119.5
N5—O1—Ho1	94.80 (14)	C17—C18—C19	120.4 (2)
N5—O2—Ho1	96.29 (14)	C17—C18—H18A	119.8
N6—O4—Ho1	94.87 (13)	C19—C18—H18A	119.8
N6—O5—Ho1	97.48 (14)	C20—C19—C23	118.2 (2)
N7—O7—Ho1	94.54 (13)	C20—C19—C18	122.0 (2)
N7—O8—Ho1	97.67 (14)	C23—C19—C18	119.9 (2)
C1—N1—C12	117.3 (2)	C21—C20—C19	119.0 (2)
C1—N1—Ho1	124.28 (16)	C21—C20—H20A	120.5
C12—N1—Ho1	117.04 (15)	C19—C20—H20A	120.5
C10—N2—C11	118.0 (2)	C20—C21—C22	119.8 (2)
C10—N2—Ho1	122.42 (17)	C20—C21—H21A	120.1
C11—N2—Ho1	119.24 (15)	C22—C21—H21A	120.1
C13—N3—C24	117.6 (2)	N4—C22—C21	122.6 (2)
C13—N3—Ho1	124.18 (17)	N4—C22—H22A	118.7
C24—N3—Ho1	116.90 (15)	C21—C22—H22A	118.7
C22—N4—C23	117.9 (2)	N4—C23—C19	122.5 (2)
C22—N4—Ho1	122.37 (16)	N4—C23—C24	117.6 (2)
C23—N4—Ho1	119.53 (15)	C19—C23—C24	120.0 (2)
O3—N5—O2	122.0 (2)	N3—C24—C16	122.4 (2)
O3—N5—O1	121.5 (2)	N3—C24—C23	118.5 (2)
O2—N5—O1	116.5 (2)	C16—C24—C23	119.1 (2)
			
O8—Ho1—O1—N5	87.34 (14)	O5—Ho1—N5—O2	−90.17 (15)
O2—Ho1—O1—N5	1.82 (13)	O1—Ho1—N5—O2	176.7 (2)
O5—Ho1—O1—N5	−77.59 (14)	N2—Ho1—N5—O2	154.63 (14)
N2—Ho1—O1—N5	159.19 (15)	N4—Ho1—N5—O2	−25.95 (15)
N4—Ho1—O1—N5	−28.91 (17)	O4—Ho1—N5—O2	−140.60 (15)
O4—Ho1—O1—N5	−132.67 (15)	O7—Ho1—N5—O2	41.35 (15)
O7—Ho1—O1—N5	46.63 (15)	N1—Ho1—N5—O2	79.75 (19)
N1—Ho1—O1—N5	121.68 (15)	N3—Ho1—N5—O2	−96.44 (16)
N3—Ho1—O1—N5	−123.61 (14)	N7—Ho1—N5—O2	66.44 (14)
N7—Ho1—O1—N5	67.27 (14)	O8—Ho1—N5—O1	−84.41 (14)
O8—Ho1—O2—N5	−80.46 (15)	O2—Ho1—N5—O1	−176.7 (2)
O5—Ho1—O2—N5	80.28 (14)	O5—Ho1—N5—O1	93.11 (14)
O1—Ho1—O2—N5	−1.84 (13)	N2—Ho1—N5—O1	−22.08 (16)
N2—Ho1—O2—N5	−33.15 (18)	N4—Ho1—N5—O1	157.34 (14)
N4—Ho1—O2—N5	154.94 (15)	O4—Ho1—N5—O1	42.69 (14)
O4—Ho1—O2—N5	41.09 (15)	O7—Ho1—N5—O1	−135.36 (15)
O7—Ho1—O2—N5	−134.50 (16)	N1—Ho1—N5—O1	−96.96 (17)
N1—Ho1—O2—N5	−131.53 (14)	N3—Ho1—N5—O1	86.84 (18)
N3—Ho1—O2—N5	116.95 (15)	N7—Ho1—N5—O1	−110.28 (15)
N7—Ho1—O2—N5	−106.88 (15)	Ho1—O4—N6—O6	−177.3 (2)
O8—Ho1—O4—N6	131.73 (13)	Ho1—O4—N6—O5	2.2 (2)
O2—Ho1—O4—N6	48.37 (14)	Ho1—O5—N6—O6	177.3 (2)
O5—Ho1—O4—N6	−1.31 (12)	Ho1—O5—N6—O4	−2.2 (2)
O1—Ho1—O4—N6	84.26 (14)	O8—Ho1—N6—O6	60 (4)
N2—Ho1—O4—N6	178.40 (15)	O2—Ho1—N6—O6	−7(4)
N4—Ho1—O4—N6	−40.73 (14)	O5—Ho1—N6—O6	−55 (4)
O7—Ho1—O4—N6	79.4 (4)	O1—Ho1—N6—O6	43 (4)
N1—Ho1—O4—N6	−137.35 (13)	N2—Ho1—N6—O6	126 (4)
N3—Ho1—O4—N6	−88.94 (14)	N4—Ho1—N6—O6	−89 (4)
N5—Ho1—O4—N6	65.15 (13)	O4—Ho1—N6—O6	127 (4)
N7—Ho1—O4—N6	126.10 (15)	O7—Ho1—N6—O6	−36 (4)
O8—Ho1—O5—N6	−96.43 (15)	N1—Ho1—N6—O6	−175 (4)
O2—Ho1—O5—N6	−127.84 (14)	N3—Ho1—N6—O6	−151 (4)
O1—Ho1—O5—N6	−72.61 (13)	N5—Ho1—N6—O6	16 (4)
N2—Ho1—O5—N6	0.99 (15)	N7—Ho1—N6—O6	23 (4)
N4—Ho1—O5—N6	144.30 (14)	O8—Ho1—N6—O4	−67.52 (16)
O4—Ho1—O5—N6	1.30 (12)	O2—Ho1—N6—O4	−133.92 (13)
O7—Ho1—O5—N6	−168.92 (12)	O5—Ho1—N6—O4	177.7 (2)
N1—Ho1—O5—N6	88.27 (15)	O1—Ho1—N6—O4	−84.47 (13)
N3—Ho1—O5—N6	75.92 (13)	N2—Ho1—N6—O4	−1.49 (14)
N5—Ho1—O5—N6	−100.29 (14)	N4—Ho1—N6—O4	143.57 (13)
N7—Ho1—O5—N6	−138.58 (13)	O7—Ho1—N6—O4	−163.38 (13)
O8—Ho1—O7—N7	1.41 (12)	N1—Ho1—N6—O4	57.33 (16)
O2—Ho1—O7—N7	90.91 (14)	N3—Ho1—N6—O4	81.45 (13)
O5—Ho1—O7—N7	133.20 (13)	N5—Ho1—N6—O4	−110.82 (14)
O1—Ho1—O7—N7	53.82 (14)	N7—Ho1—N6—O4	−104.08 (17)
N2—Ho1—O7—N7	−37.28 (15)	O8—Ho1—N6—O5	114.82 (14)
N4—Ho1—O7—N7	−178.58 (15)	O2—Ho1—N6—O5	48.43 (14)
O4—Ho1—O7—N7	58.5 (5)	O1—Ho1—N6—O5	97.88 (14)
N1—Ho1—O7—N7	−86.90 (14)	N2—Ho1—N6—O5	−179.14 (13)
N3—Ho1—O7—N7	−133.98 (13)	N4—Ho1—N6—O5	−34.08 (14)
N5—Ho1—O7—N7	72.68 (14)	O4—Ho1—N6—O5	−177.7 (2)
O2—Ho1—O8—N7	−73.77 (14)	O7—Ho1—N6—O5	19.0 (2)
O5—Ho1—O8—N7	−104.34 (14)	N1—Ho1—N6—O5	−120.32 (14)
O1—Ho1—O8—N7	−128.16 (14)	N3—Ho1—N6—O5	−96.20 (14)
N2—Ho1—O8—N7	141.16 (15)	N5—Ho1—N6—O5	71.52 (14)
N4—Ho1—O8—N7	−1.41 (16)	N7—Ho1—N6—O5	78.27 (19)
O4—Ho1—O8—N7	−173.41 (12)	Ho1—O7—N7—O9	178.5 (2)
O7—Ho1—O8—N7	−1.41 (12)	Ho1—O7—N7—O8	−2.4 (2)
N1—Ho1—O8—N7	72.67 (14)	Ho1—O8—N7—O9	−178.4 (2)
N3—Ho1—O8—N7	87.73 (16)	Ho1—O8—N7—O7	2.4 (2)
N5—Ho1—O8—N7	−100.58 (14)	O8—Ho1—N7—O9	39 (5)
O8—Ho1—N1—C1	−103.3 (2)	O2—Ho1—N7—O9	137 (5)
O2—Ho1—N1—C1	−52.2 (2)	O5—Ho1—N7—O9	148 (5)
O5—Ho1—N1—C1	73.7 (2)	O1—Ho1—N7—O9	87 (5)
O1—Ho1—N1—C1	−137.06 (19)	N2—Ho1—N7—O9	2(5)
N2—Ho1—N1—C1	−178.9 (2)	N4—Ho1—N7—O9	−142 (5)
N4—Ho1—N1—C1	20.7 (2)	O4—Ho1—N7—O9	50 (5)
O4—Ho1—N1—C1	135.79 (19)	O7—Ho1—N7—O9	−144 (5)
O7—Ho1—N1—C1	−49.29 (19)	N1—Ho1—N7—O9	−61 (5)
N3—Ho1—N1—C1	86.6 (2)	N3—Ho1—N7—O9	−82 (5)
N5—Ho1—N1—C1	−90.9 (2)	N5—Ho1—N7—O9	112 (5)
N7—Ho1—N1—C1	−77.0 (2)	O8—Ho1—N7—O7	−177.5 (2)
O8—Ho1—N1—C12	90.21 (17)	O2—Ho1—N7—O7	−79.01 (13)
O2—Ho1—N1—C12	141.32 (16)	O5—Ho1—N7—O7	−68.22 (16)
O5—Ho1—N1—C12	−92.77 (19)	O1—Ho1—N7—O7	−128.85 (13)
O1—Ho1—N1—C12	56.5 (2)	N2—Ho1—N7—O7	146.10 (13)
N2—Ho1—N1—C12	14.67 (16)	N4—Ho1—N7—O7	1.33 (14)
N4—Ho1—N1—C12	−145.71 (18)	O4—Ho1—N7—O7	−166.69 (12)
O4—Ho1—N1—C12	−30.66 (19)	N1—Ho1—N7—O7	83.15 (13)
O7—Ho1—N1—C12	144.26 (19)	N3—Ho1—N7—O7	61.82 (16)
N3—Ho1—N1—C12	−79.81 (17)	N5—Ho1—N7—O7	−104.24 (14)
N5—Ho1—N1—C12	102.60 (19)	O2—Ho1—N7—O8	98.44 (14)
N7—Ho1—N1—C12	116.57 (18)	O5—Ho1—N7—O8	109.23 (15)
O8—Ho1—N2—C10	91.0 (2)	O1—Ho1—N7—O8	48.61 (14)
O2—Ho1—N2—C10	42.1 (2)	N2—Ho1—N7—O8	−36.45 (14)
O5—Ho1—N2—C10	−49.0 (2)	N4—Ho1—N7—O8	178.79 (14)
O1—Ho1—N2—C10	17.7 (2)	O4—Ho1—N7—O8	10.8 (2)
N4—Ho1—N2—C10	−151.70 (18)	O7—Ho1—N7—O8	177.5 (2)
O4—Ho1—N2—C10	−49.29 (19)	N1—Ho1—N7—O8	−99.39 (14)
O7—Ho1—N2—C10	122.2 (2)	N3—Ho1—N7—O8	−120.72 (14)
N1—Ho1—N2—C10	173.6 (2)	N5—Ho1—N7—O8	73.22 (14)
N3—Ho1—N2—C10	−118.8 (2)	C12—N1—C1—C2	2.3 (4)
N5—Ho1—N2—C10	27.3 (2)	Ho1—N1—C1—C2	−164.1 (2)
N7—Ho1—N2—C10	106.8 (2)	N1—C1—C2—C3	−0.3 (4)
O8—Ho1—N2—C11	−96.21 (18)	C1—C2—C3—C4	−2.1 (4)
O2—Ho1—N2—C11	−145.08 (16)	C2—C3—C4—C12	2.4 (4)
O5—Ho1—N2—C11	123.82 (17)	C2—C3—C4—C5	−176.2 (3)
O1—Ho1—N2—C11	−169.45 (18)	C12—C4—C5—C6	−0.9 (4)
N4—Ho1—N2—C11	21.1 (2)	C3—C4—C5—C6	177.7 (3)
O4—Ho1—N2—C11	123.55 (19)	C4—C5—C6—C7	0.6 (4)
O7—Ho1—N2—C11	−64.97 (19)	C5—C6—C7—C8	−177.8 (3)
N1—Ho1—N2—C11	−13.61 (17)	C5—C6—C7—C11	1.8 (4)
N3—Ho1—N2—C11	54.00 (18)	C11—C7—C8—C9	−0.2 (4)
N5—Ho1—N2—C11	−159.81 (17)	C6—C7—C8—C9	179.4 (3)
N7—Ho1—N2—C11	−80.32 (18)	C7—C8—C9—C10	2.2 (4)
O8—Ho1—N3—C13	69.2 (2)	C11—N2—C10—C9	−1.2 (4)
O2—Ho1—N3—C13	−138.88 (19)	Ho1—N2—C10—C9	171.8 (2)
O5—Ho1—N3—C13	−103.1 (2)	C8—C9—C10—N2	−1.6 (4)
O1—Ho1—N3—C13	−57.9 (2)	C10—N2—C11—C7	3.3 (4)
N2—Ho1—N3—C13	19.4 (2)	Ho1—N2—C11—C7	−169.83 (18)
N4—Ho1—N3—C13	179.0 (2)	C10—N2—C11—C12	−175.1 (2)
O4—Ho1—N3—C13	−49.0 (2)	Ho1—N2—C11—C12	11.7 (3)
O7—Ho1—N3—C13	132.8 (2)	C8—C7—C11—N2	−2.7 (4)
N1—Ho1—N3—C13	85.1 (2)	C6—C7—C11—N2	177.7 (2)
N5—Ho1—N3—C13	−97.1 (2)	C8—C7—C11—C12	175.7 (2)
N7—Ho1—N3—C13	106.8 (2)	C6—C7—C11—C12	−3.9 (4)
O8—Ho1—N3—C24	−97.43 (19)	C1—N1—C12—C4	−1.9 (4)
O2—Ho1—N3—C24	54.5 (2)	Ho1—N1—C12—C4	165.5 (2)
O5—Ho1—N3—C24	90.20 (18)	C1—N1—C12—C11	177.5 (2)
O1—Ho1—N3—C24	135.39 (17)	Ho1—N1—C12—C11	−15.1 (3)
N2—Ho1—N3—C24	−147.22 (18)	C3—C4—C12—N1	−0.4 (4)
N4—Ho1—N3—C24	12.32 (17)	C5—C4—C12—N1	178.3 (2)
O4—Ho1—N3—C24	144.31 (19)	C3—C4—C12—C11	−179.8 (2)
O7—Ho1—N3—C24	−33.9 (2)	C5—C4—C12—C11	−1.1 (4)
N1—Ho1—N3—C24	−81.59 (18)	N2—C11—C12—N1	2.6 (3)
N5—Ho1—N3—C24	96.2 (2)	C7—C11—C12—N1	−175.9 (2)
N7—Ho1—N3—C24	−59.9 (2)	N2—C11—C12—C4	−178.0 (2)
O8—Ho1—N4—C22	−47.9 (2)	C7—C11—C12—C4	3.5 (4)
O2—Ho1—N4—C22	20.90 (19)	C24—N3—C13—C14	2.2 (4)
O5—Ho1—N4—C22	93.42 (19)	Ho1—N3—C13—C14	−164.4 (2)
O1—Ho1—N4—C22	44.9 (2)	N3—C13—C14—C15	−1.1 (5)
N2—Ho1—N4—C22	−148.90 (18)	C13—C14—C15—C16	−1.4 (5)
O4—Ho1—N4—C22	124.79 (19)	C14—C15—C16—C24	2.5 (4)
O7—Ho1—N4—C22	−47.88 (19)	C14—C15—C16—C17	−176.8 (3)
N1—Ho1—N4—C22	−117.5 (2)	C24—C16—C17—C18	−0.9 (5)
N3—Ho1—N4—C22	174.7 (2)	C15—C16—C17—C18	178.4 (3)
N5—Ho1—N4—C22	32.0 (2)	C16—C17—C18—C19	1.2 (5)
N7—Ho1—N4—C22	−48.50 (19)	C17—C18—C19—C20	−179.3 (3)
O8—Ho1—N4—C23	127.24 (17)	C17—C18—C19—C23	1.0 (4)
O2—Ho1—N4—C23	−163.98 (18)	C23—C19—C20—C21	0.9 (4)
O5—Ho1—N4—C23	−91.46 (18)	C18—C19—C20—C21	−178.9 (3)
O1—Ho1—N4—C23	−139.93 (16)	C19—C20—C21—C22	1.8 (4)
N2—Ho1—N4—C23	26.2 (2)	C23—N4—C22—C21	−0.3 (4)
O4—Ho1—N4—C23	−60.09 (19)	Ho1—N4—C22—C21	174.89 (19)
O7—Ho1—N4—C23	127.24 (18)	C20—C21—C22—N4	−2.2 (4)
N1—Ho1—N4—C23	57.63 (18)	C22—N4—C23—C19	3.2 (4)
N3—Ho1—N4—C23	−10.19 (17)	Ho1—N4—C23—C19	−172.13 (19)
N5—Ho1—N4—C23	−152.84 (17)	C22—N4—C23—C24	−177.2 (2)
N7—Ho1—N4—C23	126.62 (17)	Ho1—N4—C23—C24	7.5 (3)
Ho1—O2—N5—O3	−176.9 (2)	C20—C19—C23—N4	−3.5 (4)
Ho1—O2—N5—O1	3.1 (2)	C18—C19—C23—N4	176.3 (2)
Ho1—O1—N5—O3	176.9 (2)	C20—C19—C23—C24	176.9 (2)
Ho1—O1—N5—O2	−3.1 (2)	C18—C19—C23—C24	−3.4 (4)
O8—Ho1—N5—O3	178 (100)	C13—N3—C24—C16	−0.8 (4)
O2—Ho1—N5—O3	85 (4)	Ho1—N3—C24—C16	166.8 (2)
O5—Ho1—N5—O3	−5(4)	C13—N3—C24—C23	178.5 (2)
O1—Ho1—N5—O3	−98 (4)	Ho1—N3—C24—C23	−13.9 (3)
N2—Ho1—N5—O3	−120 (4)	C15—C16—C24—N3	−1.5 (4)
N4—Ho1—N5—O3	59 (4)	C17—C16—C24—N3	177.8 (3)
O4—Ho1—N5—O3	−55 (4)	C15—C16—C24—C23	179.2 (3)
O7—Ho1—N5—O3	127 (4)	C17—C16—C24—C23	−1.5 (4)
N1—Ho1—N5—O3	165 (4)	N4—C23—C24—N3	4.6 (4)
N3—Ho1—N5—O3	−11 (4)	C19—C23—C24—N3	−175.7 (2)
N7—Ho1—N5—O3	152 (4)	N4—C23—C24—C16	−176.1 (2)
O8—Ho1—N5—O2	92.30 (15)	C19—C23—C24—C16	3.6 (4)

Symmetry codes:  (i) *x*−3/2, −*y*−1/2, *z*−1/2; (ii) *x*−1/2, −*y*−1/2, *z*−3/2.

### Hydrogen-bond geometry (Å, °)

**Table:**

*D*—H···*A*	*D*—H	H···*A*	*D*···*A*	*D*—H···*A*
C1—H1A···O7	0.93	2.54	2.997 (3)	111
C3—H3A···O8^iii^	0.93	2.53	3.422 (3)	161
C9—H9A···O3^iv^	0.93	2.55	3.453 (3)	164
C13—H13A···O4	0.93	2.54	3.005 (3)	111
C21—H21A···O3^v^	0.93	2.43	3.201 (3)	140
C22—H22A···O2	0.93	2.59	3.224 (3)	126

Symmetry codes: (iii) −*x*+1/2, *y*+1/2, −*z*+1/2; (iv) −*x*+1, −*y*, −*z*+1; (v) −*x*+1, −*y*, −*z*.
